# Development of a Tunable LED-Based Colorimetric Source

**DOI:** 10.6028/jres.107.029

**Published:** 2002-08-01

**Authors:** Steven W. Brown, Carlos Santana, George P. Eppeldauer

**Affiliations:** National Institute of Standards and Technology, Gaithersburg, MD 20899-0001

**Keywords:** colorimetry, integrating sphere, light emitting diode, photometry, radiometry

## Abstract

A novel, spectrally tunable light-source utilizing light emitting diodes (LEDs) for radiometric, photometric, and colorimetric applications is described. The tunable source can simulate standard sources and can be used as a transfer source to propagate photometric and colorimetric scales from calibrated reference instruments to test artifacts with minimal increase in uncertainty. In this prototype source, 40 LEDs with 10 different spectral distributions were mounted onto an integrating sphere. A voltage-to-current control circuit was designed and implemented, enabling independent control of the current sent to each set of four LEDs. The LEDs have been characterized for stability and dependence on drive current. The prototype source demonstrates the feasibility of development of a spectrally tunable LED source using LEDs with up to 40 different spectral distributions. Simulations demonstrate that such a source would be able to approximate standard light-source distributions over the visible spectral range—from 380 nm to 780 nm—with deviations on the order of 2 %. The tunable LED source can also simulate spectral distributions of special sources such as discharge lamps and display monitors. With this tunable source, a test instrument can be rapidly calibrated against a variety of different source distributions tailored to the anticipated uses of the artifact. Target uncertainties for the calibration of test artifacts are less than 2 % in luminance and 0.002 in chromaticity for any source distribution.

## 1. Introduction

There has been a great deal of interest in the development of a stable, spectrally tunable, radiometric source for a variety of photometric, colorimetric and radiometric programs. For example, commercial integrating sphere sources typically utilize broadband sources such as incandescent lamps or Xe-arc sources with fixed spectral distributions. Colorimeters can be calibrated against these sources with minimal errors, on the order of 0.001 in chromaticity (*x*,*y*) and 1 % in luminance (*Y*). However, errors in measurements of artifacts with different spectral distributions—e.g., colored sources such as displays—using these colorimeters can be an order of magnitude larger. Ideally, these colorimeters should be calibrated using light sources with spectral distributions similar to the distributions of test artifacts measured by the colorimeters. Development of a spectrally tunable source would enable rapid calibration of these colorimeters when measuring different spectral distributions. Additional applications include evaluation of material properties under differing illumination conditions, characterization of reflective materials and displays, and calibrations of instruments that measure nonstandard spectral distributions. A spectrally tunable source using a conventional lamp and monochromator with a multi-element liquid crystal filter is currently under development [1].

Recent advances in materials and manufacture have resulted in the commercial availability of light emitting diodes (LEDs) with narrow spectral distributions, high power, and dominant wavelengths spanning the entire visible spectrum [2]. The radiometric and photometric properties of a variety of LEDs have been measured [3], and they are being further evaluated as possible standard sources [4,5]. By equipping an integrating sphere with a number of LEDs having different spectral distributions and varying the radiometric output of the various LEDs, the sphere radiance can be tuned from the blue to the red, as well as tuned to approximate CIE-defined standard illuminant distributions (e.g., Illuminant A or D65), enabling calibrations of colorimeters and spectroradiometers against different spectral distributions. Detector-based photometric and tristimulus colorimetric scales have been realized on filtered trap-detectors where spectral corrections were introduced to perform high accuracy illuminance and color measurements of sources with widely varying spectral distributions [6]. The high accuracy scales can be transferred to working standard and test meters utilizing the tunable LED source and adjusting its spectral distribution to approximate that of a specific test source. In this work, we present simulations of an idealized LED-based source and its ability to reproduce standard illuminant distributions along with the design and characterization of an LED-based integrating sphere source.

## 2. Simulations

**Fig. 1:**
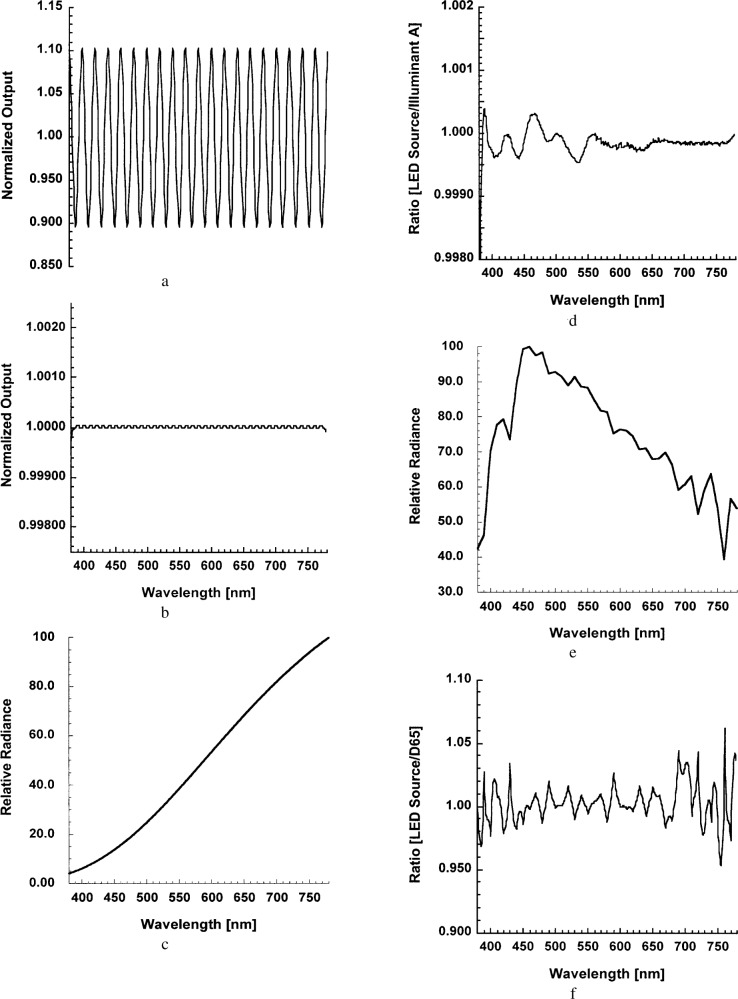
Simulations of colored source approximations for equi-energy source distribution for (a) 20 LEDs and (b) 40 LEDs over the spectral range from 380 nm to 780 nm; (c) Spectral distribution of CIE Standard Illuminant A over the range from 380 nm to 780 nm; (d) Ratio of simulated LED source distribution with 40 LEDs to Illuminant A; (e) Spectral distribution of CIE Standard Illuminant D65 over the range from 380 nm to 780 nm; (f) Ratio of simulated LED source distribution with 40 LEDs to D65.

Fig. 1, we show the results of simulations of an idealized LED source with 20 and 40 LEDs having Gaussian spectral distributions with 20 nm widths spanning the colorimetric spectral range from 380 nm to 780 nm. The simulations demonstrate that incorporation of 40 LEDs with different spectral distributions into a single unified source could enable sufficient reproduction of standard illuminant sources and possibly common display phosphor distributions.

We recently developed a prototype source to evaluate the potential development of LED-based tunable integrating sphere sources (ISS) for photometric, colorimetric and radiometric applications. The prototype ISS described in this work is equipped with a total of 40 LEDs, 4 LEDs each with 10 different spectral distributions. We describe the design and characterization of the electronic circuit as well as the aging characteristics of typical red, green and blue LEDs used in the source.

## 3. ISS Design and Layout

**Fig. 2:**
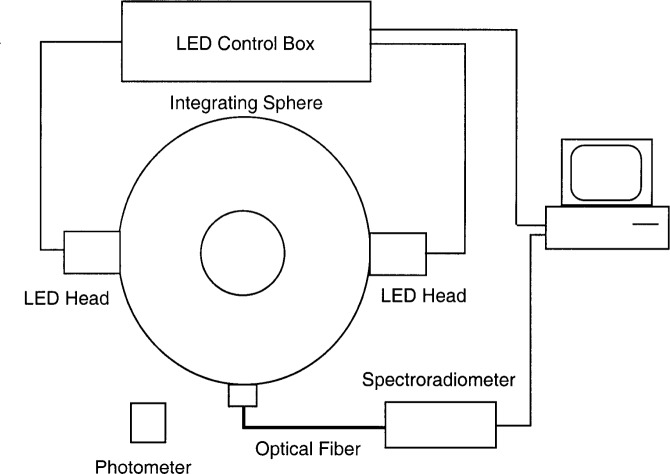
Schematic diagram of the LED source.

A schematic diagram of the LED source is shown in Fig. 2. A 10-channel electronic control box determines the current sent to each set of 4 LEDs. Twenty LEDs are included in each of two source modules that mount on either side of a commercial integrating sphere. Two LEDs in each module are connected in series and controlled with one channel in the electronic control box. The source is designed to be stable, but, because of its tunability, it is not developed as a standard calibrated source with long term reproducibility. Instead, two monitor detectors, a single channel photopic detector and a diode array spectroradiometer, are used to determine the real-time photometric or spectral radiometric output of the ISS. A computer logs the photometric or radiometric output of the monitor detector and can turn the LEDs on or off. In the future, the current to each set of LEDs will be remotely set by a computer through the General Purpose Interface Bus (GPIB).

### 3.1 Electronic Control Box

**Fig. 3:**
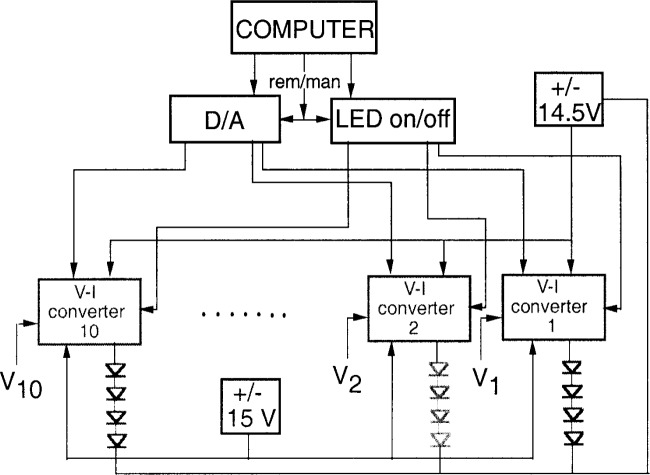
Schematic diagram of the electronic control box.

A simplified schematic diagram of the electronic control-box is shown in Fig. 3. Ten voltage-to-current (*V*-*I*) converter circuits provide a constant current to 10 sets of 4 LEDs. The current of each LED drive circuit can be switched on and off in either manual or remote control mode. Also, the input voltages, *V*_1_ to *V*_10_, of the *V*-*I* converters can be controlled in both operational modes (manual or remote). Two power supplies are used. A ± 14.5 V reference voltage source feeds the power stages of the *V*-*I* converters, while the other circuits are operated from a ± 15 V power supply.

**Fig. 4:**
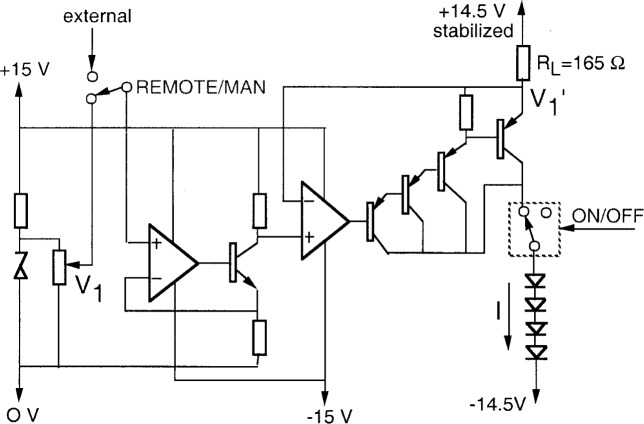
Circuit diagram of the *V*-*I* converter.

The circuit diagram of a *V*-*I* converter is shown in detail in Fig. 4. The input voltage *V*_1_ is attenuated from a Zener diode by a 10-turn potentiometer equipped with a reading dial. Alternatively, the output voltage of a D/A converter can be used in remote control mode. The input voltage *V*_1_ controls the output voltage $V_{1}^{\prime }$ through a two stage amplifier. The first stage is an inverting voltage amplifier. The second stage is a voltage follower for the load resistor (*R*_L_) that works as a current source for the serially connected LEDs. Both stages utilize analog control loops (using negative feedback from the output back to the input) to obtain high signal gain accuracy and stability. The highly stable supply-voltage of the output current source is 0.5 V lower than the + 15 V power supply of the second-stage control circuit. Consequently, $V_{1}^{\prime }$ does not have to be higher than 14.5 V. The 0.5 V difference allows $V_{1}^{\prime }$ to follow the output voltage of the first stage with a high accuracy even for very small LED current selections. The current of the LEDs can be easily switched on and off using reed relays. The TTL input of the reed relays can be controlled in both manual and remote modes. The LED current is determined by the load resistor *R*_L_ and the voltage difference between its terminals:

$$I=\frac{14.5V-V_{1}^{\prime }}{R_{L}}.$$ (1)

The LED current will be stable if the $\left(14.5V-V_{1}^{\prime }\right)$ difference and the load resistance remain constant. A high wattage resistor with a small temperature coefficient was selected for *R*_L_.

### 3.2 Optical Head Design

**Fig. 5:**
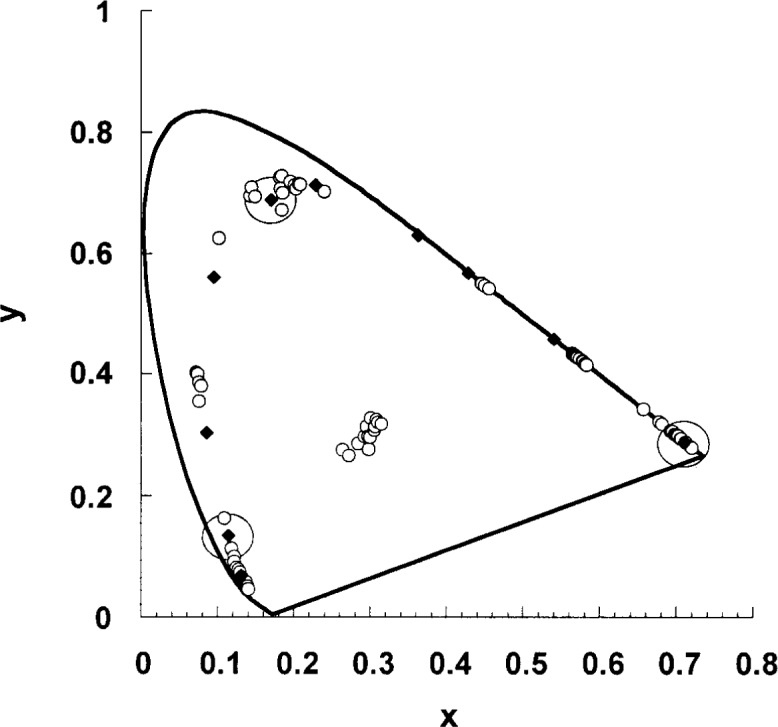
Chromaticity coordinate diagram with the chromaticity coordinates of a variety of LEDs. Closed diamonds represent chromaticity coordinates of LEDs installed in the source. Circled symbols are chromaticity coordinates of red, green and blue LEDs discussed in the text. Uncertainties are reported in Ref. [2].

The LED source module was designed to fit onto existing ports in a commercial integrating sphere. The source module was designed to enable rapid change of a particular set of LEDs within a module, or for the entire module to be easily changed. Two sets of source modules were fabricated. One set of source modules was equipped with LEDs having spectral distributions ranging from the blue to the red. The spectral distribution and chromaticity coordinates of a number of LEDs have been measured [2]. Their chromaticity coordinates are shown in Fig. 5. From these LEDs, 10 sets of 4 LEDs with chromaticity coordinates varying from the blue to the red were mounted in the source module. Their chromaticity coordinates are also shown in Fig. 5.

### 3.3 Monitor Detectors

The integrating sphere source has one detector port. There are two interchangeable monitor detectors, a single element silicon detector with a photopic filter for monitoring the photometric output of the source and a commercial fiber-coupled diode-array spectroradiometer for continuously monitoring the spectral output of the source over the range from 350 nm to 860 nm.

## 4. Source Characterization

The electrical and optical properties of the LED source have been characterized. Each is discussed in detail below.

### 4.1 Electronic Characterization

**Fig. 6:**
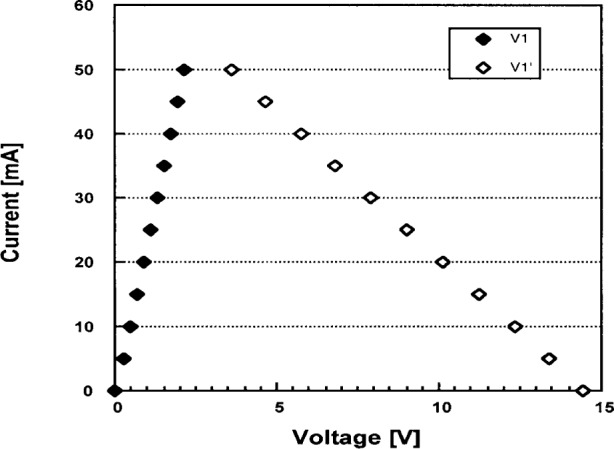
Range of the control voltages of the *V*-*I* converter versus LED current.

**Fig. 7:**
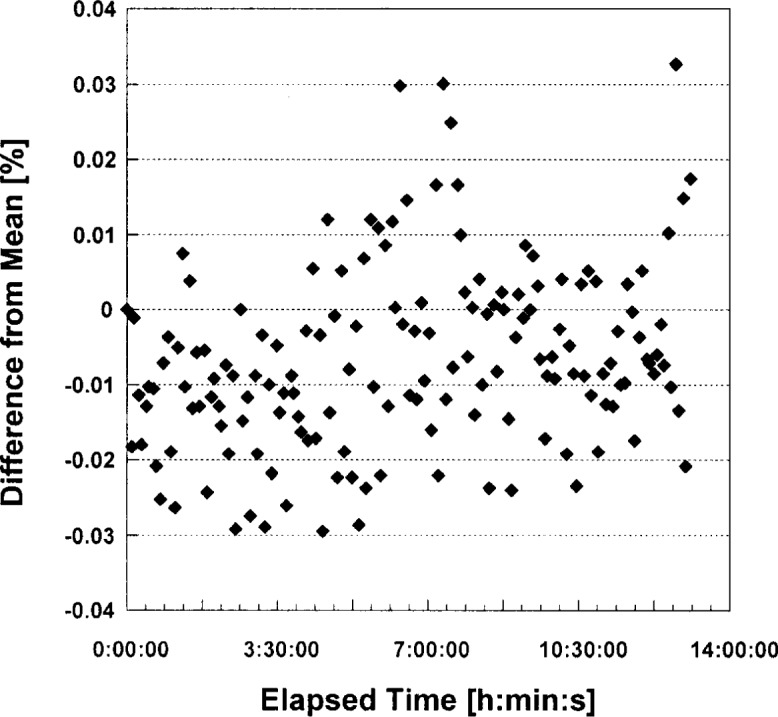
Stability of the output current of the *V*-*I* converter to a set of LEDs.

The range of the control voltages *V*_1_ and $V_{1}^{\prime }$ for a load resistor of about 200 Ω is shown in Fig. 6. As the graph shows, the relation between the LED current and the control voltages is linear. The LED current can be regulated from 0 mA to 50 mA with a resolution of approximately 0.01 mA. Figure 7 shows the results of the stability test of the voltage-to-current converter. The output current to the selected LEDs was stable to better than 0.03 % of the set point (in this case 35 mA) over 14 h. Similar studies have been extended to 100 h with similar stability. No long term drifts in the current have been observed over a total operational time of 500 h.

### 4.2 Optical Characterization

The optical characteristics of representative red, green and blue LEDs used in the source are presented. The chromaticity coordinates of these LEDs are circled in Fig. 5. Each set of 4 LEDs was aged for 100 h, and their chromaticity and luminance monitored for the next 50 h of operation. For each set, the chromaticity was stable to within 0.001 in *x* and *y*, well within our target chromaticity uncertainty of 0.002. The measured luminance from the green and blue LEDs was stable to within 0.5 %, also well within our target uncertainty of 2 %, while the red luminance changed by 4 %. Further aging studies of the red LEDs are currently under way to establish the longer term stability of those LEDs. The source is stable to better than 0.2 % in all cases over the duration of several hours, the typical duration for a calibration.

**Fig. 8:**
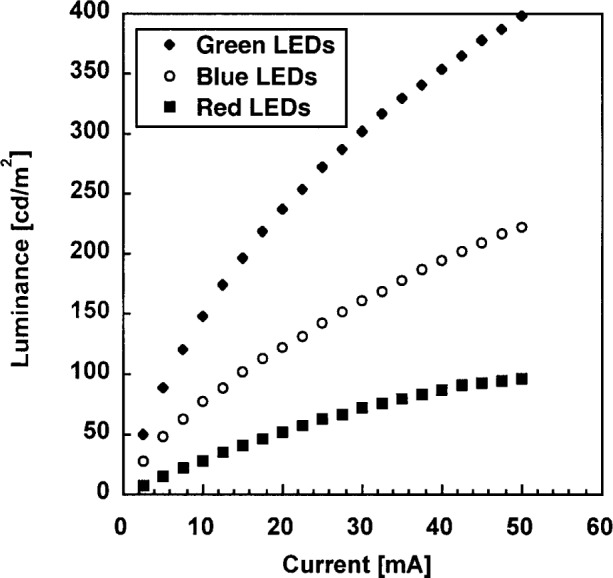
The relationship between the luminance and the drive current of a representative set of red, green, and blue LEDs (circled chromaticity coordinates in Fig. 5).

The relationship between the luminance and the drive current of each set of LEDs was established, as shown in Fig. 8. The normalized relationship was very similar for the three sets of LEDs. The luminance of each set of LEDs approaches the luminance of a CRT display set to saturated red, green, and blue colors. With 50 mA supplied to all ten sets of LEDs, the source luminance was approximately 1000 cd/m^2^ and its integrated radiance was approximately 500 μW/cm^2^/sr. Incorporating newly developed higher intensity green and red LEDs, the source output can easily be doubled or tripled. The radiometric and photometric output of the source is comparable to that of a conventional lamp-illuminated integrating sphere source in the visible spectral region [7]. Note that the LED source has negligible output in the infrared spectral region. In contrast, the output from a lamp-illuminated integrating sphere source with a color temperature of 3000 K peaks in the infrared at 1000 nm and has significant radiant flux in the spectral range from 1000 nm to 2500 nm.

**Fig. 9:**
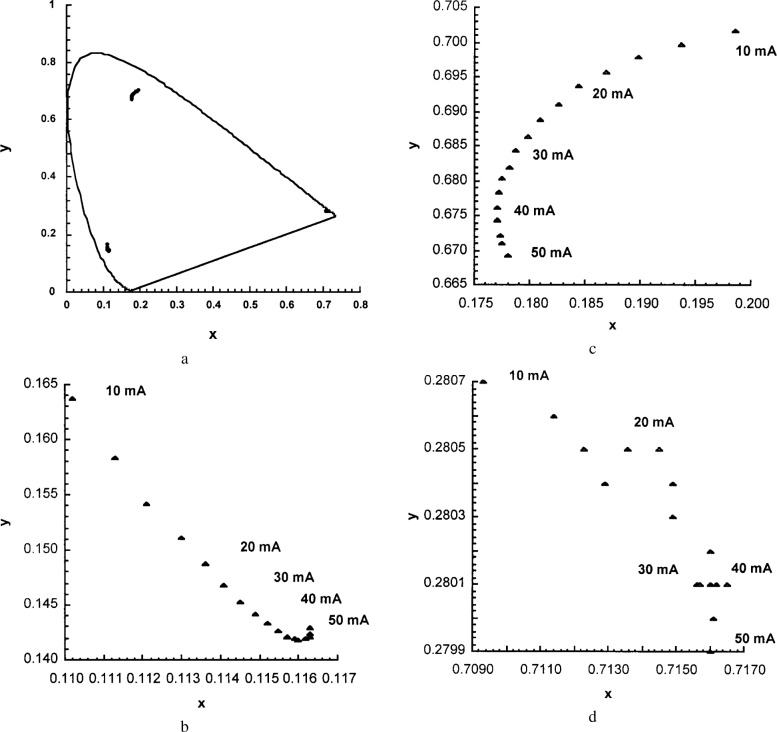
The chromaticity coordinates of red, green, and blue LEDs as a function of drive current (a). Expanded view of blue (b), green (c), and red (d) LED chromaticity coordinates as a function of drive current.

The chromaticity coordinates of the different LEDs were a strong function of drive current, as shown in Fig. 9. These changes arise from shifts in the spectral distribution of the LED with drive current. In developing the algorithm to approximate a specific source distribution, the spectral shift of each set of LEDs must be measured as a function of drive current and included in the model.

## 5. Summary

We have developed a prototype spectrally tunable source utilizing 40 LEDs with 10 different spectral distributions. A voltage-to-current control circuit was designed and implemented, enabling independent control of the current sent to each set of LEDs. The LEDs have been characterized for stability and dependence on drive current. The prototype source demonstrates the feasibility of development of a high-brightness, spectrally tunable LED source with independent control of up to 40 different sets of LEDs. Simulations demonstrate that such a source would be able to approximate the spectral distributions of standard illuminants over the visible range—from 380 nm to 780 nm—with errors on the order of 2 % in luminance and 0.002 in chromaticity. The tunable LED source will be useful in transferring photometric and colorimetric scales from reference instruments to test artifacts with a minimum increase in uncertainty for specific spectral distributions.
